# Examining the presence and effects of coherence and fragmentation in the Gulf of Maine fishery management network

**DOI:** 10.1007/s10113-024-02328-y

**Published:** 2024-12-06

**Authors:** Derek Katznelson, Antonia Sohns, Dongkyu Kim, Evelyn Roozee, William R. Donner, Andrew M. Song, Jasper R. de Vries, Owen Temby, Gordon M. Hickey

**Affiliations:** 1 Department of Sociology, The University of Texas Rio Grande Valley, Edinburg, TX USA; 2 Department of Natural Resource Sciences, McGill University, Montreal, QC Canada; 3 Department of Political Science, The University of Texas Rio Grande Valley, Edinburg, TX USA; 4 Faculty of Arts and Social Sciences, University of Technology Sydney, Ultimo, NSW Australia; 5 Landscape Architecture and Spatial Planning Cluster, Wageningen University, Wageningen, Netherlands; 6 School of Earth, Environmental, and Marine Sciences, The University of Texas Rio Grande Valley, One West University Blvd., Brownsville, TX 78520 USA

**Keywords:** Transboundary fishery governance, Risk, Gulf of Maine, Networks, Collaboration

## Abstract

Natural resource management networks cohere due to mutual dependencies and fragment, in part, due to the perceived risks of interaction. However, research on these networks has tended to accept coherence a priori rather than problematizing dependence, and few studies exist on interorganizational risk perception. This article presents the results of a study operationalizing these concepts and measuring the distribution of three types of dependence (capital, legitimacy, and regulatory) and two types of perceived risk (performance and sanction) among nearly fifty stakeholder groups and organizations participating in the management of fisheries in the binational Gulf of Maine. The analysis reveals an organizationally diverse network with several stakeholder types participating, with communications clustered binationally, with low levels of perceived risk in interacting, and interdependencies cohering the network. The types of interorganizational dependence present varied across dyadic relationships, but legitimacy dependence, based on shared understandings that organizations should work together, was the most present and had the largest effect on collaboration-oriented network traits. Sanction risk was more common than performance risk but had the most substantial negative effect. The results suggest an opportunity for additional studies of interorganizational dependance and perceived risk to operationalize and measure the sources of network coherence and fragmentation and their effect on collaboration.

**Supplementary Information:**

The online version contains supplementary material available at 10.1007/s10113-024-02328-y.

## Introduction

It is axiomatic in the natural resource management (NRM) literature that organizations network based on functional dependencies to address environmental challenges. The failures that arise through this networking process are a central focus of the field in the hopes of better understanding what goes wrong. Coherence—the centripetal forces that bring about these networks—and fragmentation—the centrifugal forces that shape professional associations—are among the constitutive social facts of NRM. They enable and hamper the collaborative processes necessary for collectively managing and adapting to complexity (Feist et al. 2020; Imperial 2005; Stern 2018).

Given this, it is notable that little systematic research has been done on the types of dependencies that drive NRM network coherence. The different types of dependencies may lead to divergent network outcomes. Similarly, limited attention has been given to the perceived risks that fragment networks of civil servants, who know each other or each other’s organization, and engage strategically based on subjective assessments of the pitfalls of engagement. Case studies on ecosystem-based management (EBM) initiatives have addressed this, to be sure, but not systematically with conceptual operationalization or sample-based data collection. The content of network coherence and fragmentation in NRM, and the extent to which they drive or inhibit collaborative processes, is an area in need of further exploration.

A growing literature examines the formation and management of transnational natural resource networks addressing environmental challenges across administrative borders (Healy et al. 2014; Temby and Stoett 2017). Transnational contexts are particularly challenging due to differing regulatory frameworks, objectives, and monitoring and enforcement agencies (Cisneros-Montemayor et al. 2021; Palacios-Abrantes et al. 2020). Consequently, the needed input, process, and result are far from clear, oftentimes leading to high risk perception (Wondolleck and Yaffee 2017, ch. 2). High risk perception may result in retentive behavior, delays, and conflicts that can hamper collaboration. For example, a global analysis of marine species that cross multiple Exclusive Economic Zones (EEZs) found that catches of transboundary fish species are declining at a faster rate than in non-transboundary species, indicating there are widespread problems of collaboration in fishery management (Palacios-Abrantes et al. 2020).

One of the places where transboundary collaboration is key for sustainable fishery management is the Gulf of Maine (GOM), where a network of collaborative governing bodies has been institutionalized across jurisdictional boundaries. The enduring history of the binational GOM fishery management network provides an instrumental case for analyzing the dynamics of interorganizational dependence and risk perception, and their effect on collaboration.

### Networks, dependence, and risk

In NRM networks, the knowledge and information required in decision-making are distributed within and across bureaucratic agencies, especially in the context of shared regulatory jurisdictions and organizational responsibilities (de Arruda Leite and Buainain 2013; Imperial 2005; Song et al. 2019). Working in networks means breaking down organizational boundaries to communicate and coordinate with partners at other organizations, which potentially improves information sharing and relationship trust (van Meerkerk and Edelenbos 2018). Although organizations maintain autonomy, they are still impacted by their relationships with partners in the network environment and cannot operate in isolation from one another (Carlsson and Berkes 2005; Delerue 2005). Relationships within a network have varying levels of dependence that may become contested through competing interests (Bodin and Crona 2009) and may be positively associated with collaborative antecedents like belief in one’s abilities to make a difference or inhibitors like conflicting goals (Cinner and Barnes 2019; Furnari 2016). Interdependence is a driver of collaboration between partners and shapes the structure of the resulting network and flows of information and knowledge (de Arruda Leite and Buainain 2013).

Collaboration also exposes partners to the perceived risk^1^ that other groups may monopolize a network’s goals and plans to capture the collaborative process (Amy 1987; Hickey et al. 2021; Walker and Hurley 2004). Partners rely on one another to achieve their joint objective, but there are inevitable uncertainties in the outcome and the possibility of failure. As a result, a partner’s perceived risk of each network relationship is determined by the partner’s ability to mitigate uncertainties. This may occur by exerting control over the situation or by exercising trust in a partner to assuage fear of uncertainties (Das and Teng 2001; de Arruda Leite and Buainain 2013). Perceived risks based on past experiences with partners may inform whether a partner will engage in future collaborative relationships and therefore can be a force of network fragmentation. Perceived risk has been found to have an inverse relationship with features of collaborative interaction, such as self-efficacy, and a positive relationship with conflict (Hodgins et al. 2006; Niemi et al. 1991; Witte 1992). The concept of self-efficacy is distinguished in studies of political contexts between internal efficacy and external efficacy. Internal efficacy refers to one’s assessment of their competence to understand and participate in decisions, and external efficacy captures people’s perceptions of powerfulness and powerlessness in the political context (Craig et al. 1990; Morrell 2003; Niemi et al. 1991). In NRM, Cinner and Barnes (2019) refer to it as “agency” and Norman (2017) as community “self-determination.” Cinner and Barnes (2019) argue that agency or efficacy of a network actor is a social factor determining the resilience of a socio-ecological system. However, the extent to which the cohering network traits (i.e., dependence types) and the fragmenting traits (i.e., perceived risk) affect collaboration-oriented network traits like self-efficacy and goal conflict in transboundary NRM contexts is unclear.

### Gulf of Maine transboundary fishery management

The interior waters of the GOM foster diverse ecosystems that make it a “sea within a sea,” with different temperatures and salinity from the Atlantic Ocean (Hildebrand et al. 2002, 423). The GOM’s unique character has made it one of the world’s most biologically productive bodies of water, supporting economically and ecologically critical fish species (Hildebrand et al. 2002). The GOM offers an important case study of transboundary interorganizational collaboration due to the region’s history of resource overexploitation and the continued social and economic importance of the fishery resource systems, including the highly valuable American lobster, which have necessitated sustained collaborative governance between state, provincial, and federal partners in Canada and the USA (Le Bris et al. 2018; Shibles 1994) who manage shared and migratory fish stocks spanning their jurisdictional boundaries (Koubrak and VanderZwaag 2020; Russell and VanderZwaag 2010).

In 1984, a maritime boundary dividing the GOM between the USA and Canada was established by a decision of the World Court (Chircop et al. 1995). Subsequently, in 1989, the Governors of Maine, New Hampshire, and Massachusetts, and the Premiers of Nova Scotia and New Brunswick agreed to form the Gulf of Maine Council on the Marine Environment (GOMC) and asserted the importance of “the cooperative pursuit of consistent policies, initiatives and programs” for the “sustainable development and use” of the “precious public natural resources... that form part of an overall ecosystem that transcends political boundaries” (Gulf of Maine Council on the Marine Environment 1991, appendix). The GOMC was created to oversee the inter-provincial-state partnership agreement and continues to focus on convening partners, organizing partners and resources, supporting projects in the GOM, and educating the public regarding the GOM (Hildebrand et al. 2002; Wondolleck and Yaffee 2017).

The GOMC along with other transboundary cooperative partnerships manages the competing interests in the shared fishery resources. The network of collaborative governing bodies includes the federal agencies of Canada and the USA, as well as state and provincial governments, First Nation and tribal governments, along with non-governmental organizations. Inter-jurisdictional organizations have also been developed to manage certain fish species, conduct cooperative scientific studies, or govern issues related to GOM fishery resources, such as the North Atlantic Salmon Conservation Organization, the Northwest Atlantic Fisheries Organization, the International Commission for the Conservation of Atlantic Tunas, the New England Fishery Management Council, the Atlantic States Marine Fisheries Commission, and the Gulf States Marine Fisheries Commission (Koubrak and VanderZwaag 2020). These transboundary management structures are adaptable, usually do not have regulatory or enforcement mechanisms, and seek to jointly develop policy and share information (Hildebrand et al. 2002). The enabling structure of this transboundary network focuses on creating a shared understanding and coordination through a cascade of information, priorities, and relationships (Wondolleck and Yaffee 2017). Critically, they depend on collaboration between partners to achieve their stated goals and the organization’s mission (Hickey et al. 2021).

Despite the existing network of collaborative governance, the GOM suffers from non-compliance with fisheries regulations and the legacy of rapidly depleted fish stocks including cod, yellowtail flounder, and haddock (Pudden and VanderZwaag 2007). Further, the unofficial management of stocks, through suggested versus enforceable allocation rules, may make many of these management bodies less resilient to the effects of rapid environmental change (Palacios-Abrantes et al. 2020; Sumaila et al. 2020). Climate change is already exacerbating fishery management challenges in the GOM as sea temperatures increase faster than 99% of the world’s oceans, the oceans acidify, and species potentially shift ranges (Bricknell et al. 2021; Pershing et al. 2015). Shifting species’ ranges in a transboundary governance setting could threaten management objectives such as gear requirements or conservation measures (Palacios-Abrantes et al. 2020). In the GOM, where management mechanisms differ on either side of the border, local changes to fish presence and abundance could significantly affect collaboration between partners in the governing network (Palacios-Abrantes et al. 2020; Sumaila et al. 2020). A deeper understanding of network fragmentation and coherence is needed to improve the collaborative performance of the GOM transboundary fishery network.

This paper aims to measure perceived levels of interorganizational dependence and risk to determine their presence and examine their effect, in the GOM transboundary fishery network, on collaboration-oriented network traits, namely, external efficacy and goal conflict.

## Materials and methods

The research methods consisted of a survey instrument (IRB-19-0254) distributed to participants in fishery management in the Gulf of Maine from August to October 2019. Forty-nine organizations were selected from online partner lists, news articles, and key informant feedback, paying attention to ensure reasonable inclusion and depth. It was not intended or necessary for the organization list to be exhaustive. The survey was distributed by email to people professionally affiliated with the 49 organizations and by phone and email to decision-makers who volunteered to share the link with potential respondents. The email explained the project scope and included a link to the Qualtrics survey platform.

Respondents were asked a series of biographical questions, including the organization for which they work (although they were not asked to represent this organization in their response), role in that organization, years at that organization, work postal code, and presented with a list of other organizations participating in the region’s fishery management. They were then asked to select which organizations they communicate with in their role, and follow-up questions about their relationships with these organizations. This resulted in a dataset with a dyadic structure and variables measured on respondent-target organization ties. A total of 2460 potential respondents were collected and received invitations, with 102 surveys completed. This yielded data on 890 respondent-target organization dyads. All responses were anonymous with no identifying personal information included in the data set.

### Measuring dependence and risk

The respondents used the 5-point Likert scale (strongly agree, agree, neither agree nor disagree, disagree, and strongly disagree) to measure the variables operationalized for interorganizational dependence and risk perception. We distinguished among four types of interorganizational dependence known to drive network coherence: (1) legitimacy, (2) epistemic, (3) financial, and (4) regulatory. *Legitimacy* occurs when diverse stakeholder groups are incorporated in deliberations (Lockwood et al. 2010). It has the potential to increase a network’s resources and survival (Meyer and Rowan 1977). *Epistemic dependence* exists when the knowledge needed to perform network functions is distributed unevenly across network partners, such as scientists, local resource users, or bureaucrats (Bouwen and Taillieu 2004; Eisenhardt and Santos 2012; Grant and Baden-Fuller 2004). Similarly, *financial resources* are critical to determine whether an organization has the necessary equipment or funding to operate and survive. In a common-pool resource community, if an organization is not integrated into the economic market, it may have failed to network with financiers and funding (Bodin and Crona 2008). *Regulatory dependence* exists in situations where organizations rely on the government-granted authority of other organizations to make collectively binding decisions for shared management and policy objectives (Hickey et al. 2023).

To determine which questions on dependence to retain for analysis, Exploratory Factor Analysis (EFA) was used on the 9 survey items for dependency measures. The result shows that there are three factors whose values exceed the value of 1. Regulatory and legitimacy variables are consistently clustered while financial and epistemic dependence variables loaded up together. Thus, we chose one item from each of the informational and financial dependence categories and created a new factor, “capital dependence.” Organizations in this high knowledge environment could readily exchange funding and knowledge to create value. This further supports the idea of “intellectual capital,” where knowledge resources are valuable like financial resources in situations of high uncertainty (Martín-de-Castro et al. 2011).^2^ Regulation explained 40% of the variance, capital dependence for 13% and legitimacy for 11% (Table 1).

**Table 1:** Dependence components based on varimax rotation matrix

	Variable	Definition	Survey Measurement	EFA value
Regulatory dependence	REGD1	Reliance on the government-granted authority of other organizations to make collectively binding decisions for shared management and policy objectives (Hickey et al. 2023)	Our organization depends on this organization to enforce, enact, comply, or design regulations and policy	0.887
	REGD2		Without this organization’s authority to make collectively binding decisions, it would be difficult for us to meet our objectives	0.866
Capital dependence	CAPD1	The distribution of knowledge, data, and financial resources to create specialized value for the network	This organization provides us important funding	0.798
	CAPD2		I get information from this organization that I would not have known to ask for	0.551
Legitimacy dependence	LEGD1	The inclusion of multiple levels of stakeholders, which reinforces an organizations position as participants (Barnaud and van Paassen 2013)	Working with this organization is expected as part of an inclusive fisheries management process	0.815
	LEGD2		Working with this organization prevents management problems from arising down the road	0.870

The survey had a total of five items for measures risk perception, which was further categorized into three types: (1) relational risk, (2) performance risk, and (3) regulatory and sanction risk (Anderson et al. 2014). The factor analysis shows these five survey items have two common factors whose eigenvalues exceed the value of 1. The first cluster was clearly observed for two performance risk items. However, one item of relational risk and another item for regulatory and sanction risk form another cluster. Accordingly, EFA generates two measures of risk perceptions: performance risk and sanction risk. Perception of performance risk accounts for 34% and perception of sanction risk accounts for 23% of the variance, respectively (Table 2).

**Table 2:** Risk components based on varimax rotation matrix

	Variable	Definition	Survey Measurement	EFA value
Performance risk	PR2	The probability and consequence that alliance objectives are not achieved despite satisfactory cooperation (Das and Teng 2001)	The outcome is usually positive when I deal with this organization	0.786
	PR1		I question this organization's competence	0.753
Sanction risk	SR1	The probability and consequence of a partner exposing the firm to sanctions from a third party by failing to comply with rules (Anderson et al. 2014)	A decision made by this organization can significantly impact my organization	0.749
	SR2		The actions of this organization may expose my organization to regulatory sanctions if relevant rules are not followed	0.787

To measure external efficacy, we adapted the efficacy scales of Craig et al. (1990). The respondent was asked two questions on how they feel about their ability to interact with each organization they reported communicating with. EFA shows that these two variables have one common factor as only one factor has an eigenvalue greater than 1. A question measuring internal efficacy was used to control for the respondents’ self-perception of their empowerment. As for the goal conflict, we follow the strategy of Song et al. (2019). The respondents were asked if the communicating organization’s goals conflict with their organization. The variable adopts the 5 Likert scale (Table 3).

**Table 3:** Efficacy components

	Variable	Definition	Survey measurement
External efficacy	EE1	The belief that a participant has the meaningful power to express themself in a politicized structure (Morrell 2003)	This organization accepts input from my organization into how it functions
	EE2		Generally speaking, this organization has lost touch with my organization
Internal efficacy	IE	The personal belief in the competence of oneself in a political context (Craig et al. 1990)	I feel that I have a good understanding of the important issues facing fisheries in the Gulf of Maine
Goal conflict	GC	The absence of perceived alignment of organizational goals in an interorganizational context (Provan and Kenis 2008)	This organization's goals conflict with those of our organization

### Descriptive and inferential analysis

Analysis consisted of descriptive procedures to map relationships among network participants and inferential procedures to demonstrate the effects of dependence and risk perception types on collaboration-related network traits, namely, goal conflict and external efficacy. Relationship mapping was performed for the three dependence types and two risk perception types. In this procedure, organizations with which respondents reported communicating were grouped into the following stakeholder categories: regional governmental organizations, U.S. federal agencies, Canadian federal agencies, U.S. state agencies, Canadian provincial agencies, Indigenous tribes, research institutions, environmental non-governmental organizations (NGOs), and fisher groups. The scores that each respondent assigned each organization they reported communicating with were standardized on a − 1 to 1 scale and averaged across organizations in each of the nine stakeholder categories. The result was a stakeholder category score for each dependence and risk perception type for each respondent. These were then grouped by the stakeholder category of the respondent’s reported employer to provide stakeholder category-to-category scores for the risk perception types.

Hierarchical regression was used to analyze variation of these variables. This inferential approach is suitable when the analysis seeks to highlight the effects of specific predictor sets and when variables are highly correlated (Lima et al. 2019; Song et al. 2019; Temby et al. 2017). It involves the construction of predicative models consisting of predictor sets of one or more variables that are entered sequentially into the hierarchical regression models with an ordering scheme based on a theoretical rationale. Each predictor set explains the variation not explained by the previously entered predictor sets, with the increment in R squared values. Standardized coefficients were used to assess the effects on the dependent variable of each individual predictor placed into the predictor sets.

Hierarchical regression models were created for each of the two dependent variables: external efficacy and goal conflict. Goal conflict also included the first dependent variable, external efficacy, as a predictor. The analysis controlled for variation among respondents’ affiliated organization category, the organization category of the groups with which respondents reported communicating, respondent’s internal efficacy, and individual level variation (using criterion scaling). Internal efficacy was included to control for dispositional attributes that may contribute to external efficacy, a relationally defined concept describing one’s efficacy toward external referents (Ostrander et al. 2017). Participant and target organizations were aggregated into the following categories: (1) regional governmental organizations, (2) U.S. federal agencies, (3) Canadian federal agencies, (4) U.S. state agencies, (5) Canadian provincial agencies, (6) Indigenous tribes, (7) research institutions, (8) environmental non-governmental organizations (NGOs), and (9) fisher groups. These categories were then dummy-coded using Canadian provincial agencies as the reference category.

Because each survey respondent is represented in several respondent-target organization dyads, the data had a repeated measure characteristic. If included in the analysis, the survey respondents with valid responses would have required more than 100 dummy-coded variables. Pedhazur (1977) and Gibbons and Sherwood (1985) recommend an alternative data-analytic approach, called *criterion scaling*, for encoding data with repeated measures. This approach was applied to create a single predictor using each respondent’s mean score on the dependent variable as the predictor value for all the organizations on which the respondent answered questions. For a further elaboration of this approach with dyadic survey data, see Temby et al. (2017).

Interaction variables were created between each of the two risk perception types and internal efficacy and all three dependence types. A total of eight interaction terms were created. The predictor sets were defined and entered sequentially into the hierarchical regression models using the following general logic: (1) control variables, (2) independent variables, and (3) interactions. Table 4 shows the specific order, the rationale for the order, and the dependent variable of each model.

**Table 4:** Summary of the hierarchical regression predictor sets and the order in which they entered each of the two regression model

Predictor sets in order entered	Logic for ordering of predictor set	External efficacy	Conflict
Participant organization category	Codes the most general way of classifying survey participants by jurisdiction of agency they work for, irrespective of target agency they relate to	1	1
Internal efficacy (IE)	Scales the personal belief in the competence of oneself in a political context (Craig et al. 1990). Controls for agent disposition, rather than structural environment	2	2
Criterion-scaled participants predictor	Codes individual participants to control for individual differences in rating relationships with individual agencies	3	3
Target organization category	Codes the jurisdiction of the specific agency that is a target for trust development and communications for an individual participant	4	4
Legitimacy dependence (LEGD)	Assesses the function of an organization for inclusivity for multiple levels of stakeholders, which reinforces their position as participants (Barnaud and van Paassen 2013)	5	5
Capital dependence (CAPD)	Identifies an organization who distributes knowledge, data, and financial resources to create specialized value for the network. An important function of public networks being the flexibility and speed to transmit that across organizational borders (Lockwood et al. 2010)	6	6
Regulatory dependence (REGD)	Identifies an organization’s ability to enforce policies or legally binding constraints to behavior of agents in the network, which can be important to the management of common-pool resources (Nie 2008)	7	7
Sanction risk (SR)	Assesses the level that an organization in a partnership perceives that the partner organization could cause sanctions to be put upon the respondent’s organization (Anderson et al. 2015)	8	8
Performance risk (PR) (PR2 is reverse coded)	Assesses the level of perceived risk for unsatisfactory performance in the selected organization (Das and Teng 2001)	9	9
Sanction risk interactions (SR*IE, SR*LEGD), SR*CAPD, SR*REGD)	2-way interactions between Sanction Risk and Internal Efficacy and dependence components, entered after the relevant main effects have been accounted for	10	10
Performance risk interactions (PR*IE, PR*LEGD), PR*CAPD, PR*REGD)	2-way interactions between Performance Risk and Internal Efficacy and dependence components, entered after the relevant main effects have been accounted for	11	11
External efficacy (EE2 is reverse coded)	Assesses the belief that the participant has the meaningful power to express himself or herself in a politicized structure after accounting for internal efficacy, dependencies and risks (Morrell 2003)	DV	12
Goal conflict	Assesses the impact of internal efficacy and all types of dependence and perceived risk on interagency goal conflict	–	DV

### Limitations

The structure of the survey and mode of distribution limited the range of stakeholders from whom data could be gathered, or about whom respondents could report working with. Because respondents were asked follow-up questions about other organizations, including too many would lengthen the survey for well-connected respondents and potentially lead to higher respondent attrition. Thus, some regional multi-jurisdictional organizations relevant to fisheries or aquatic habitats in the study region were excluded, like the Atlantic States Marine Fisheries Commission (ASMFC), International Commission for the Conservation of Atlantic Tunas, International Joint Commission, and the Northwest Atlantic Fisheries Organization. The study’s specific focus on international transboundary professional fishery connections meant that organizations likely to engage in strictly domestic interactions were also not prioritized for inclusion in the survey. One example is the ASMFC, whose statutory mandate extends to subnational coordination only. The consequences of these exclusions are most likely to be reflected in Fig. 2, potentially understating the connectedness of these regional organizations. This is a limitation of following a purposive sampling strategy; however, we employed several measures to strengthen the reliability and internal validity of our results. This included defining the measures of our study before initiating data collection, pre-testing our survey instrument to reduce the potential for bias, and consulting with local experts at different stages of the study design, including the list of organizations that were included in the survey (see also Temby et al. 2015; Song et al. 2019, 2020; Roozee et al. 2024).

Of greater significance is the inherent bias of email survey–based studies to prioritize commercial and offshore fishing policymakers and other stakeholders and actors over recreational anglers, charter boat fleet, Indigenous fishers, boat crew, and shoreside stakeholders like subsistence fishers and processing plant workers. The recent NOAA Fisheries (2023) *Equity and Environmental Justice Strategy*, and subsequent implementation plan for the Northeast region, prioritizes research and outreach to these underserved communities. Among the activities it mandates is the identification of and knowledge coproduction with them through a variety of means, like partnering with community organizations to find the underserved community members, hosting listening sessions, and identifying barriers to involvement with management. The narrower scope of this present study, on the more professional-bureaucratic part of the fishery management network, means that this important part of the region’s fishery system is underrepresented.

## Results

### Respondent profile

**Fig. 1:**
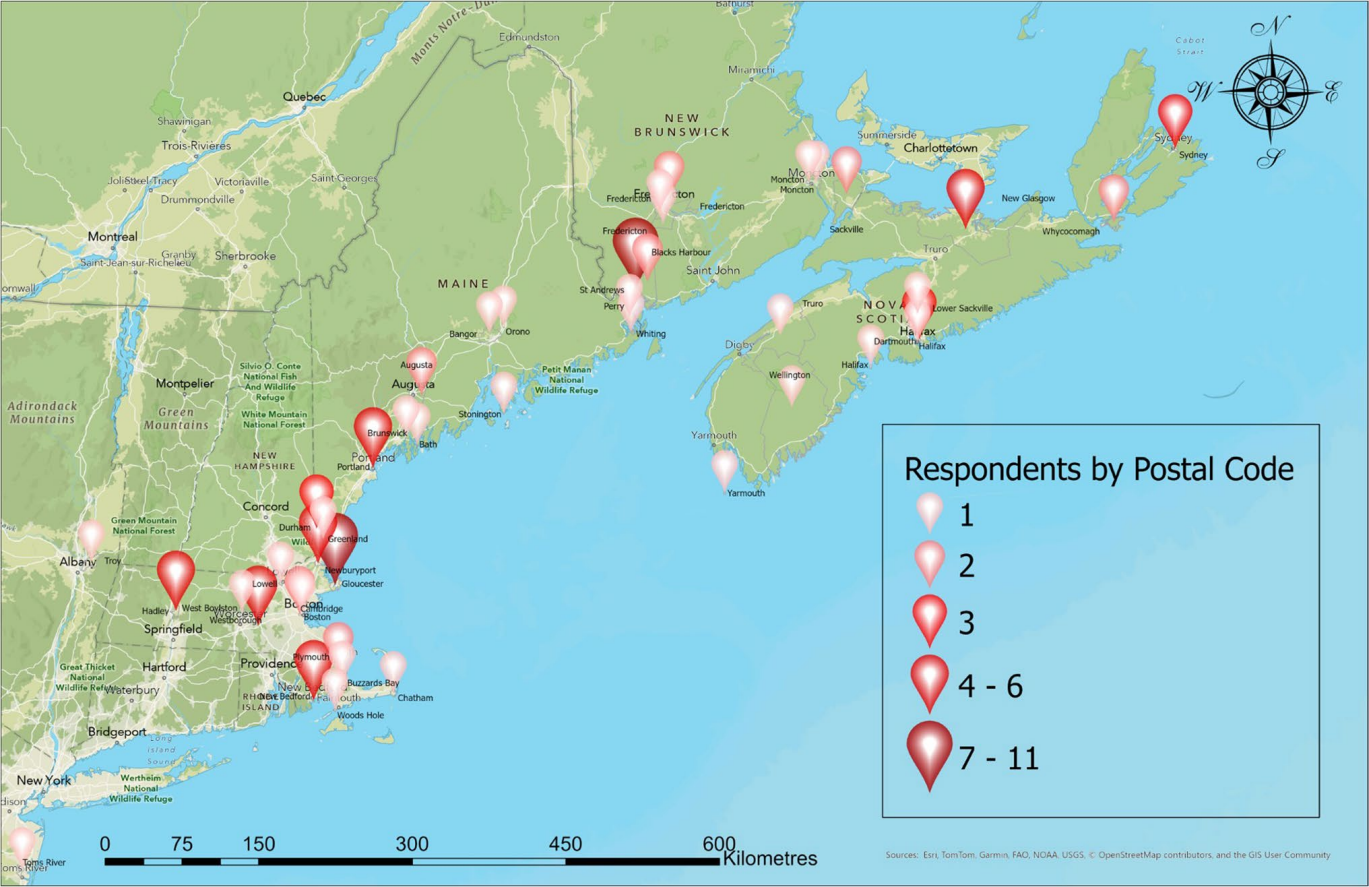
Respondent geography by postal code

Table 5 shows the distribution of respondents by category of affiliated organization. Proportion of respondents from national and subnational U.S. and Canadian governments was roughly equal. The survey received fewer respondents from regional government, universities, tribes, and fisher associations. It also found that most of the respondents work in policy, management, or research (Table 6). Based on postal codes provided by survey respondents, most worked in the bordering provincial and state jurisdictions in the GOM, with concentrations major cities and fishing ports (see Fig. 1), with Boston, Gloucester, Portland, Halifax, and St. Andrews particularly represented.

**Table 5:** Survey response by organization category (*n* = 102)

Organization category	Percent of respondents
Canadian Provincial	21.6%
US Federal	20.6%
US State	17.6%
Canadian Federal	15.7%
Research	11.8%
Regional	6.9%
Fisher Groups	3.9%
Indigenous Tribes	2%

**Table 6:** Survey respondent role type

Role type	Percent of respondents
Natural Resource Management	29%
Policy, Regulation and Administration	21%
Research (Natural Science)	21%
Other	14%
Monitoring, Compliance and Enforcement	13%
Fisher	2%

### Communication patterns among organization categories

**Fig. 2:**
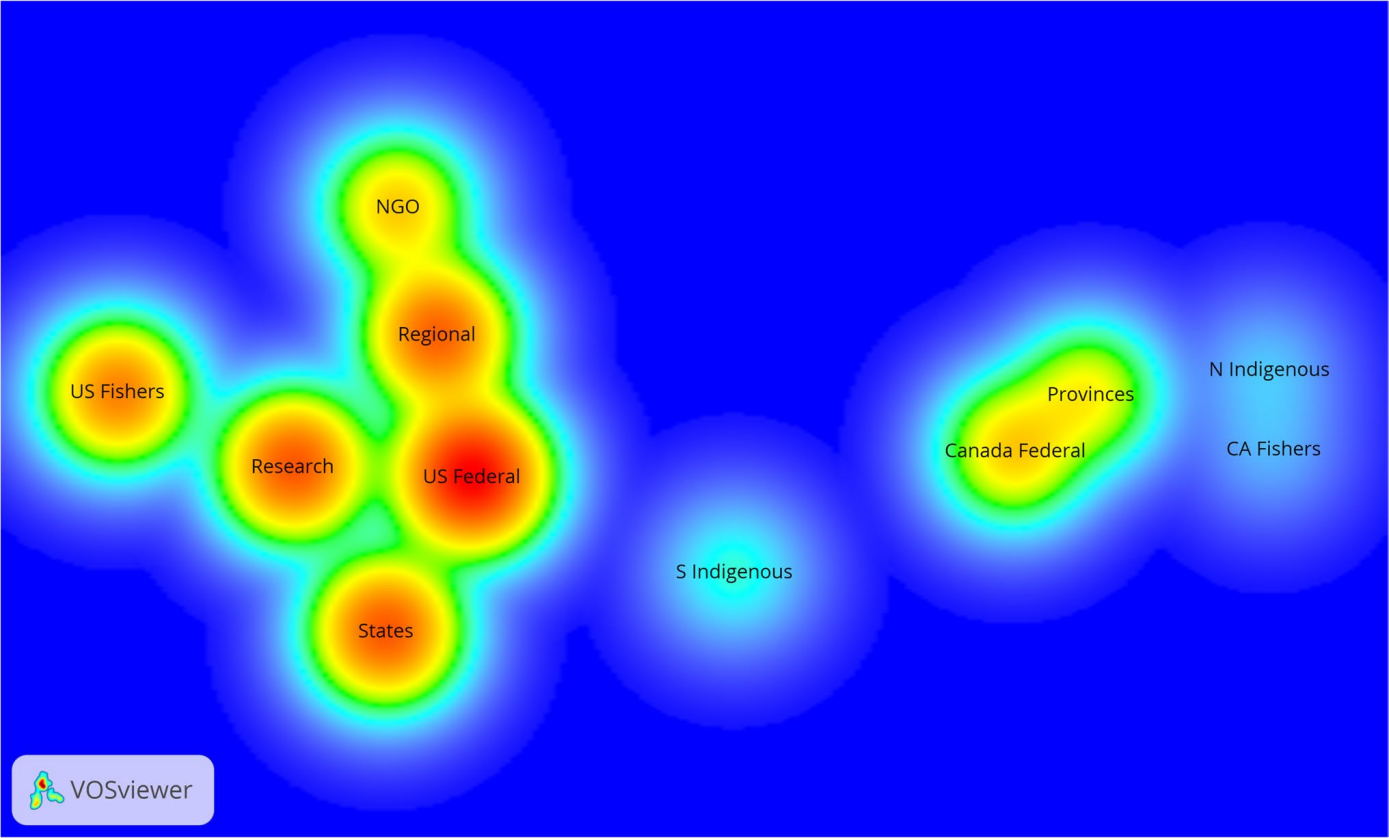
Density visualization of communicative networks among survey respondents, grouped by organizational categories. (Note: dyadic *n* = 890; normalization used was association strength, 3:1 attraction/repulsion, 1.5 resolution format; Visualization tool: Vos Viewer 1.6.19)

A distance-based density visualization was created with VOSviewer to depict the relationship patterns across respondents’ organizational affiliations and contacts, grouped by organizational category (see Fig. 2). Node color denotes the reported communicative level links in the dyadic data of interorganizational communication reported by respondents with home organization with another organization (i.e., the redder and larger the node, the greater number of communication links between an organization in each category receive from other organizations). The distance between nodes indicates the frequency of communication across categories, such that closely located nodes imply a higher frequency of communication between them according to survey respondents. The categories for Indigenous tribal groups and fisher organizations were further separated into categories for each spatial side of the Canadian-U.S. border. Although the survey could not comprehensively map the interorganizational network, grouping the categories enables us to see which types of organizations are communicated with the most, by whom, and where the communicative clusters occur.

Based on our survey sample, U.S. federal agencies are centrally located, with substantial connections to research organizations, the U.S. states, and the regionally scaled fishery organizations. However, most notable is the communicative gulf between the two sides of the national border. Organizations located in Canada communicate with others in Canada, not much with those in the USA. The sole exception is the Canadian (federal and provincial) communications with Indigenous organizations on both the north and the south sides of the national border.

### Presence and distribution of risk perception and dependence types

**Fig. 3:**
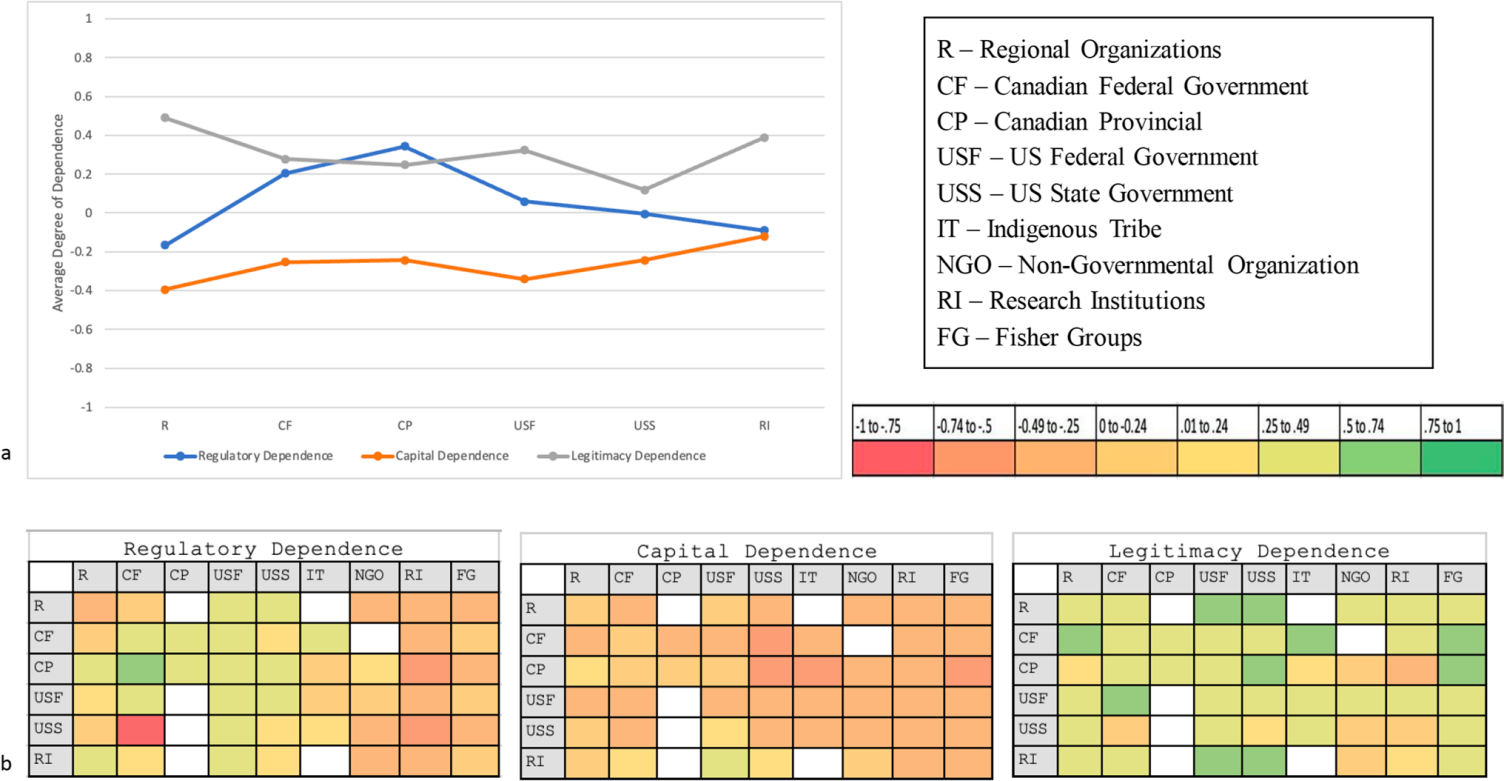
**a** Average rating of dependence by respondent organization groups comprising the Gulf of Maine fisheries policy network. **b** The average rating of regulatory dependence, capital dependence, and legitimacy dependence, toward target agency groups reported by survey respondents. The left column indicates the respondent’s home organization, and the top row indicates the target agency. Color codes indicate the average degree of dependence of survey responses. Green coding indicates high dependence, while red coding indicates low dependence, existing on a scale from − 1 to 1 (low to high)

Figure 3 shows the organizational categories with the highest reported legitimacy dependence by respondents from other organizations were U.S. federal and state agencies, and fisher groups. Indigenous groups were scored especially high by respondents from Canadian federal agencies. Both NGOs and research institutions had low reported legitimacy dependence.

**Fig. 4:**
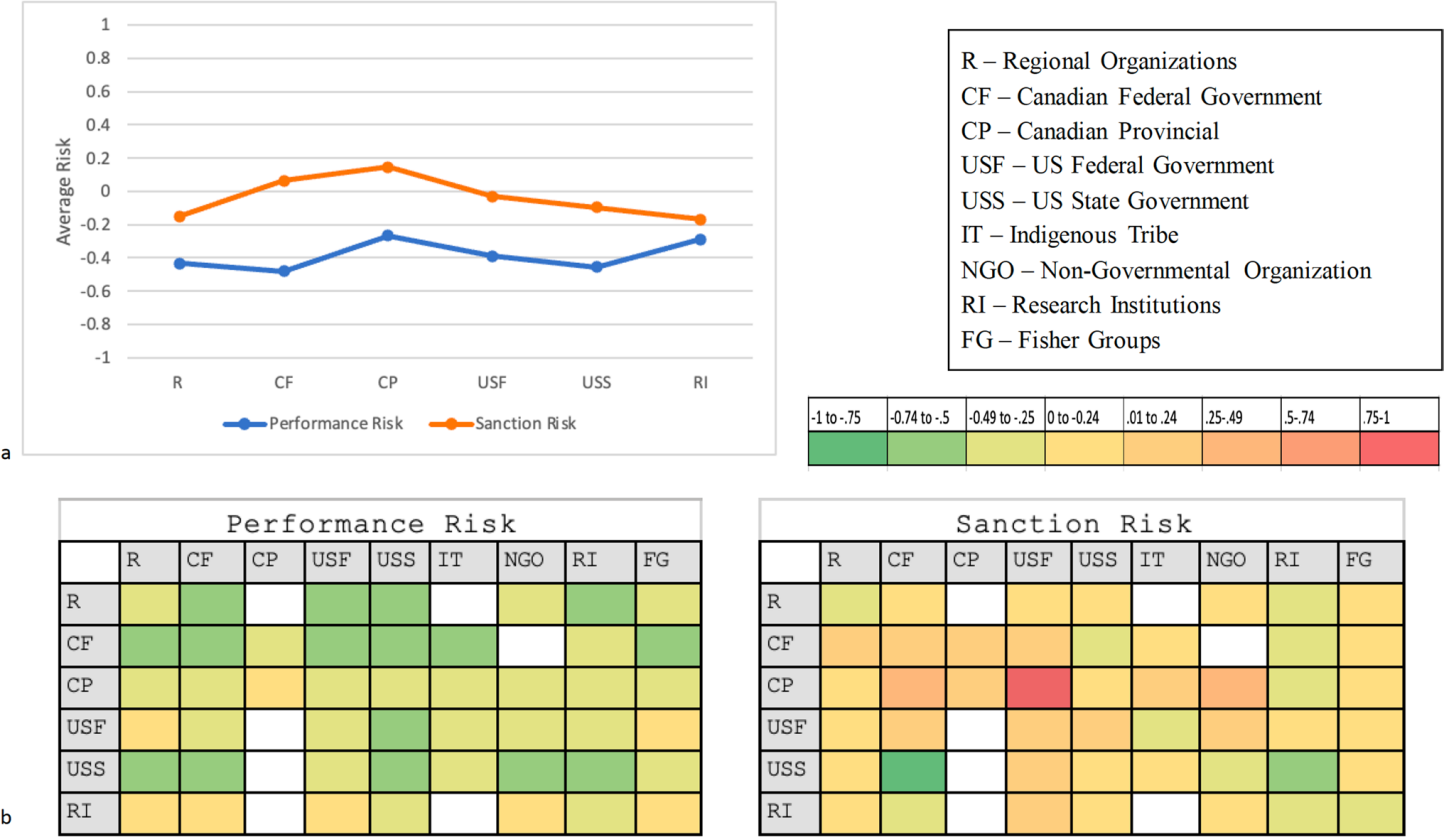
**a** Average rating of risk by respondent organization groups comprising the Gulf of Maine fisheries policy network. **b** The average rating of performance risk and sanction risk toward target agency groups reported by survey respondents. The left column indicates the respondent’s home organization, and the top row indicates the target agency. Color codes indicate the average degree of dependence of survey responses. Green coding indicates low risk, while red coding indicates high risk, existing on a scale from − 1 to 1 (low to high)

Figure 4 shows the distribution of the risk types by organization category. Performance risk across respondent organizational categories was negative overall; perceived sanction risk was higher across all categories. The U.S. federal government was reported to have the highest degree of sanction risk associated with engagement, while research institutions had the lowest in the response network. Respondents from Canadian provincial government agencies reported the highest perceived sanction risk from interacting with other agencies.

### Effect of risk and dependence on external efficacy and goal conflict

**Fig. 5:**
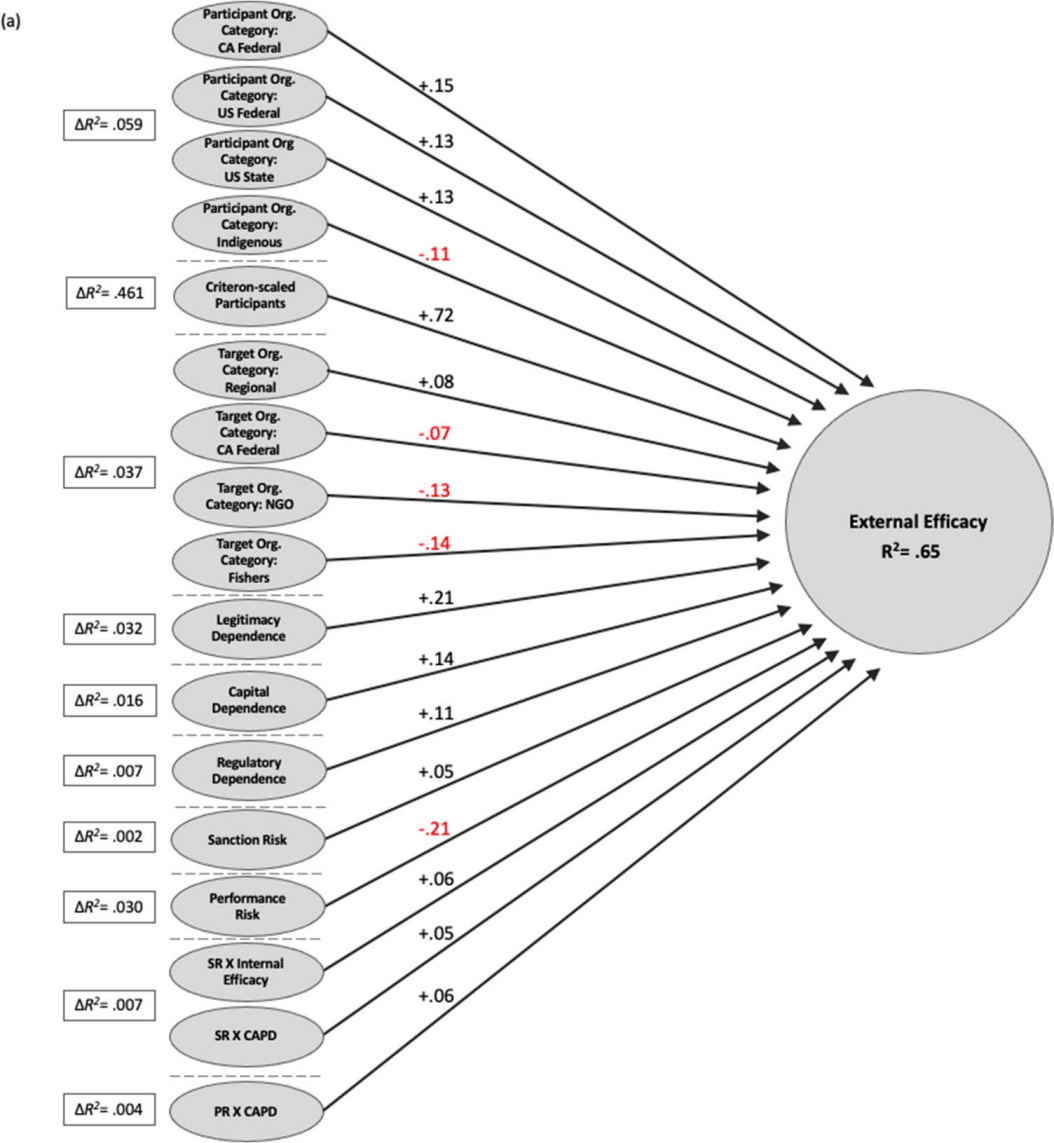

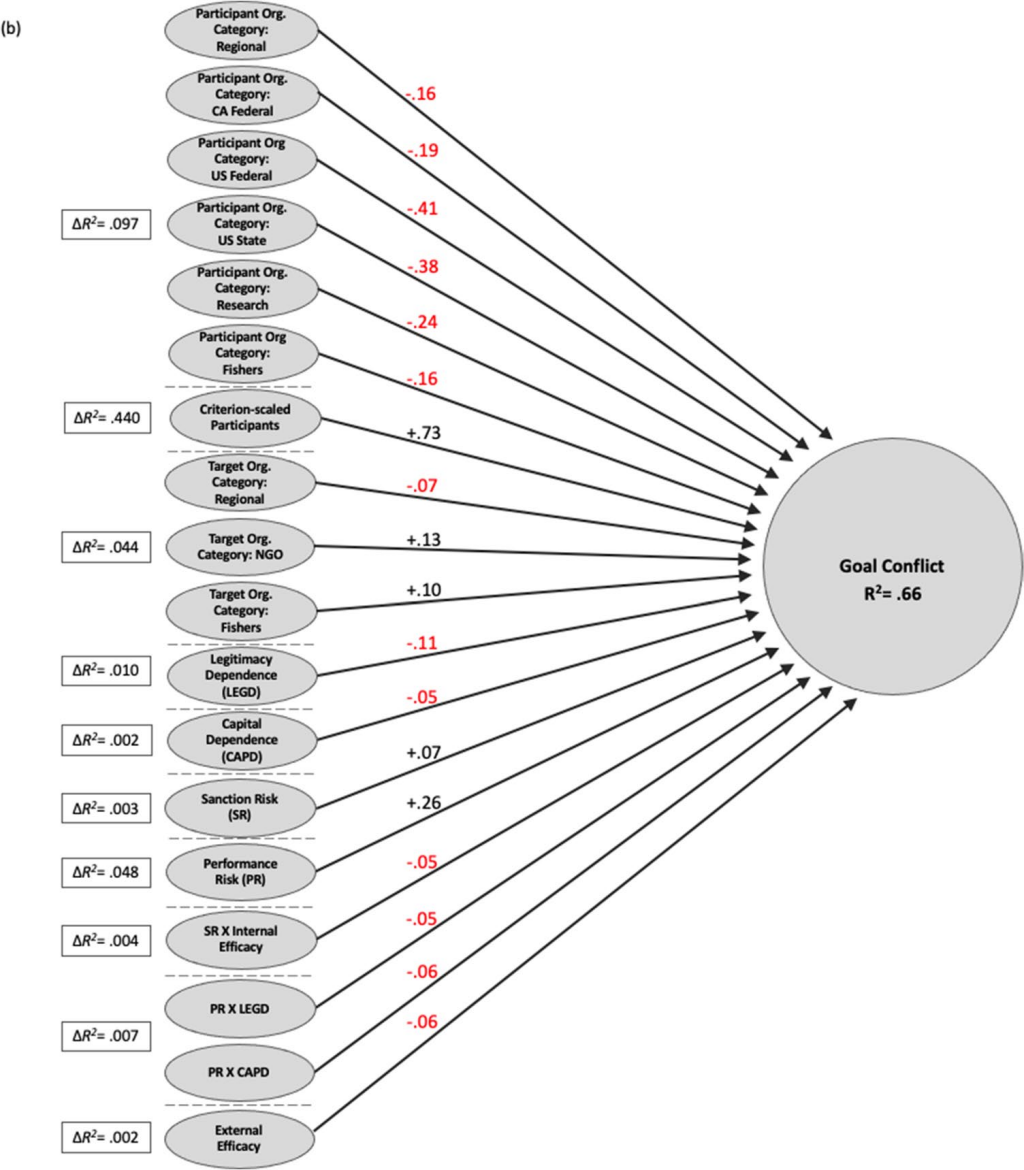
Summary of significant hierarchical regression relationships for predicting impact on (**a**) external efficacy and (**b**) conflict. The hierarchical predictor sets are separated by short, dotted lines and the change in R^2^ associated with the addition of that predictor set to each regression model is shown in solid-line boxes. Note: contribution of predictor set shown as ΔR^2^; path coefficients are standardized regression coefficients; only significant (*p* < .01) relationships are shown

Figure 5a and b present the results from hierarchical regression models for external efficacy and goal conflict. Each predictor set was depicted only if the added variable was significant at a 0.05 level or lower. Because hierarchical regression is a cumulative process, the figures display change in R^2^ values. The number on the arrow represents the standardized beta coefficient with red indicating a negative coefficient value and black indicating a positive one. Total changes in coefficient of determination (R^2^) predicted by the predictor sets were placed within the box: external efficacy with 0.65; conflict with 0.66. See Tables S1 and S2 for the full output.

Figure 5a details that all risk and dependance types predict external efficacy. Performance risk was strongly negatively associated with external efficacy. Legitimacy dependence had the largest effect on external efficacy among the three dependence types. The weak positive relationship between sanction risk and external efficacy indicates that feeling that another organization can affect a respondent’s organization through, potentially, regulatory sanctions is associated with a feeling that the same organization is interested in and accepts inputs from the respondent’s organization. In other words, the relationship suggests a degree of reciprocal influence. The interaction between sanction risk and internal efficacy indicates that this effect is stronger among respondents reporting higher levels of internal efficacy. The interaction of capital dependence with performance risk and sanction risk indicates that the effect of capital dependence on external efficacy is higher when the other forms of risk are elevated.

Figure 5b excludes regulatory dependence as a predictor for conflict. All other dependence and risk types, and the addition of external efficacy, have a statistically significant effect on goal conflict in this case study. Perceived risk, sanction risk and performance risk, raise goal conflict between organizations. In contrast, capital dependence, legitimacy dependence, and external efficacy lower conflict. Sanction risk negatively interacts with internal efficacy to produce conflict. As internal efficacy increases, the effect of sanction risk on conflict lessens. Performance risk negatively interacts with legitimacy and capital dependence. As performance risk increases, the presence of these two types of dependence mitigates the effect of performance risk on conflict. External efficacy is negatively associated with conflict. Respondents with high levels of external efficacy are more likely to report a consensus of goals, and less conflict, with partner organizations.

## Discussion and conclusion

This article has used a survey of participants in the public management network for the Gulf of Maine fishery to examine the network traits that cohere the network (i.e., interorganizational dependence) and those that potentially fragment it (i.e., perceived risk). This required that these concepts be disaggregated into dimensions, operationalized, and analyzed for alignment with underlying constructs. It has long been axiomatic that the normative basis of networks is “complementary strengths” (Powell 1990, 300) or “mutual dependence” (Agranoff and McGuire 2001, 314), but the types of strengths have so far been underspecified or not subject to systematic analysis (see, also, Isett et al. 2011; O’ Toole Jr 1997). For example, in a paper on water policy networks, Bressers et al. (1994, 4) list the dependencies that bring actors together as “authority, resources, and knowledge.” Although we found authority to be one of these cohering dependencies, resources and knowledge did not appear as individual types in our analysis. Further, we found legitimacy dependence to be the most prevalent type bringing about collaborative traits in the GOM fishery management network, underpinned by shared understandings of who should be involved in the process (legitimacy).

There are, of course, a multitude of facts other than perceived risk that may make collaborative NRM networks fail. But the relationship challenges contained within the large-scale management networks have been well documented. For example, Layzer’s (2008) study of the failures of U.S. ecosystem–based management (EBM) focused largely on the problem of goal conflict resulting from suspicion and divergent values among network participants. Wondolleck and Yaffee’s (2017, 188) work on marine EBM highlighted the problem of busy professionals with scarce time struggling to justify time spent on “side-of-the-desk” interorganizational initiatives when they do not know that they will yield results. Sanction risk and performance risk can be major problems that make interorganizational NRM initiatives underperform, despite the substantial reasons for coming together. In the GOM context, we found that risk perception levels are low in general, especially performance risk. Sanction risk—the subjective potential exposure to problems from interacting with another organization—was also low, although less so.

Overall, our results suggest that the network exhibits binationally clustered communications, low levels of perceived risk in interactions, and a shared understanding that organizations should work together. (It is unclear whether including additional multi-jurisdictional organizations as survey options would have made the two sides of the border appear more communicative on transboundary fishery issues.) The available evidence also provides insights to potential causes of binational fragmentation. One is the high level of sanction risk that respondents from Canadian provincial agencies reported for U.S. federal agencies. Another is the relatively low levels of regulatory and legitimacy dependence by respondents from the U.S. state governments for the Canadian federal government, low levels of capital dependence by respondents from the Canadian federal and provincial agencies for the U.S. state agencies, and the absence of communications reported by respondents from U.S. federal or state agencies for the Canadian provincial agencies. In other words, it appears that respondents from Canadian provincial agencies consider it risky to contact U.S. federal agencies; respondents from U.S. state agencies do not feel they need to interact much with the Canadian federal government; and respondents from Canadian provincial and federal agencies do not feel they need to interact much with U.S. state agencies. These are findings that warrant further policy and management attention.

The predictive findings indicate that interorganizational dependency is positively associated with the external efficacy of the survey respondents reporting the dependency. In other words, the belief that one’s organization relies on another organization for something it needs increases one’s belief that the other organization is responsive to these needs. Of the three dependence types, the positive effect on external efficacy was the greatest for legitimacy dependence. Perceived performance risk had the opposite effect, lowering the respondent’s feeling of external efficacy toward an organization that is perceived as disappointing and incompetent. This is consistent with Wondolleck and Yaffee’s observations about binational marine ecosystem–based management initiatives in another U.S.-Canadian transboundary area, the Salish Sea region. They showed that turnover in the British Columbia government agencies led to shifting memberships of important task forces, which “strained any sense of a common transboundary agenda” (Wondolleck and Yaffee 2017, 30).

Conversely, sanction risk shows a small positive effect on external efficacy. This is understandable given that the survey instrument measures organizations that opt to interact and communicate with each other, less those that avoid doing so. It is likely that the sampling method selects for efficacious participants who may see interaction with organizations (even those interactions that are potentially perilous) as necessary to conduct business and meet organizational objectives. This reasoning is supported by the interaction between internal efficacy and sanction risk on external efficacy, which indicates that efficacious people report more external efficacy when sanction risk is present.

Perceived performance risk was the predictor variable most associated with increasing interorganizational goal conflict. Fortunately for the Gulf of Maine fishery management case, performance risk was generally low across our sample. Sanction risk had a small effect on goal conflict but interacted negatively with internal efficacy to lessen it. Thus, the interaction effect between these two variables operated to produce collaboration-enhancing outcomes across both models. Legitimacy dependence was the variable most associated with moderating conflict. The feeling that another organization that the respondent communicates with is *expected* to be engaged with, and that doing so will prevent problems down the road, resulted in less conflict with that organization.

The dependency and risk interactions in both models are also noteworthy. Higher perceived risk was associated with a greater effect of capital dependence in producing collaboration-oriented network traits. This was shown for both risk types in amplifying external efficacy and for performance risk in attenuating goal conflict. Higher levels of performance risk also increased the effect of legitimacy dependence in attenuating goal conflict.

These findings point to the primacy of interorganizational dependence—a concept too often taken for granted in NRM network theory—for cohering networks and enabling collaboration. Rather than assuming its existence and function when a network exists, it should be investigated to determine what types are present and what types are missing. As displayed here, the differential presence and effect of dependence is an empirical question that may vary across contexts.

There is also a need for further work to refine the measures of perceived risk and dependence in NRM networks. Important questions include what is the relationship between perceived risk types and multidimensional trust? Are there other measurable dependence types and perceived risk types that are conceptually and empirically distinct from those presented in this study? Recent work by Hickey and colleagues proposes another risk type relevant to transboundary NRM, namely, relational risk (Hickey et al. 2021, 2023; Sohns et al. 2021). Its relationship to collaboration, or how to measure it, is not yet clear.

Other areas of future research this study highlights the potential utility of are as follows: What are the effects of dependence and perceived risk on other collaboration-oriented concepts and measures? And, perhaps most importantly, what can network leaders do to manage risk so that it is facilitative of collaboration (as this study has shown that limited sanction risk can be) rather than a hindrance? An emerging literature has developed on NRM management strategies, boundary-spanning leadership, and management control tools (Hickey et al. 2023; Klijn et al. 2010; van Meerkerk and Edelenbos 2020). There exists great opportunity to connect research on management practices to the management of perceived risk and dependence so that NRM networks can organize and operate more collaboratively.

## Supplementary Information

Below is the link to the electronic supplementary material.

Supplementary file1 (DOCX 71 KB)

## Data Availability

Data will be made available on request.

## Footnotes

1 Perceived risk is defined as the probability and the consequences of unfavorable outcomes occurring, such as failed collaboration in an alliance (Das and Teng 2001, 2004; Delerue 2004).

2 For a similar categorization, see Dietsch et al. (2021). These authors distinguish three sources of power in conservation partnerships, namely authority, resource-based, and discursive legitimacy.
