# Supplementary material for: Examining the presence and effects of coherence and fragmentation in the Gulf of Maine fishery management network
Source: Reg Environ Change. 2024 Dec 6;25(1):3. doi: 10.1007/s10113-024-02328-y (PMC11624248; doi:10.1007/s10113-024-02328-y)
Supplement: Supplementary file 1 — Supplementary file1 (DOCX 71 KB) [file 10113_2024_2328_MOESM1_ESM.docx]

**ELECTRONIC SUPPLEMENTARY MATERIAL**

**Appendix**

**Table S1**: Hierarchical Regression Analysis: **External Efficacy**

| Hierarchical regression model summary ^a^ | | | | | | | |
| --- | --- | --- | --- | --- | --- | --- | --- |
|  |  | R^2^ change statistics | | | | | |
| Model | Predictor set entered | Model R^2^ | R^2^ Change | *df p*redictors | *df* residual | F-test | P |
| 1 | Participant’s Organization Category | 0.0596 | 0.0596 | 7 | 882 | **7.99** | **0.0000** |
| 2 | Internal Efficacy (IE) | 0.0604 | 0.0008 | 8 | 881 | **7.08** | **0.0000** |
| 3 | Criterion-scaled Participants | 0.5216 | 0.4612 | 9 | 880 | **106.59** | **0.0000** |
| 4 | Target Organization Category | 0.5583 | 0.0367 | 17 | 872 | **64.82** | **0.0000** |
| 5 | Legitimacy Dependence (LEGD) | 0.5898 | 0.0315 | 18 | 871 | **69.56** | **0.0000** |
| 6 | Capital Dependence (CAPD) | 0.6058 | 0.0160 | 19 | 870 | **70.37** | **0.0000** |
| 7 | Regulatory Dependence (REGD) | 0.6124 | 0.0066 | 20 | 869 | **68.65** | **0.0000** |
| 8 | Sanction Risk (SR) | 0.6141 | 0.0017 | 21 | 868 | **65.78** | **0.0000** |
| 9 | Performance Risk (PR) | 0.6436 | 0.0295 | 22 | 867 | **71.18** | **0.0000** |
| 10 | SR Interactions (SR×IE, SR×LEGD, SR×CAPD, and SR×REGD) | 0.6505 | 0.0069 | 26 | 863 | **61.78** | **0.0000** |
| 11 | PR Interactions (PR×IE, PR×LEGD, PR×CAPD, and PR×REGD) | 0.6545 | 0.0040 | 30 | 859 | **54.25** | **0.0000** |
| Model coefficients | |  |  |  |  |  |  |
|  |  | Unstandardized  coefficients | | Standardized  coefficients | Correlations | | F-test |
| Model | Individual predictor | β | Std. error | β | Part | sr^2^ | P |
| 1 | Participant’s Organization Category: Regional | -0.0140 | 0.1044 | -0.0068 | -0.0044 | 0.0000 | 0.8931 |
|  | Participant’s Organization Category: CA Federal | 0.4468 | 0.1271 | 0.1483 | 0.1148 | 0.0132 | **0.0005** |
|  | Participant’s Organization Category: US Federal | 0.2237 | 0.0968 | 0.1314 | 0.0755 | 0.0057 | **0.0210** |
|  | Participant’s Organization Category: State | 0.2722 | 0.1069 | 0.1256 | 0.0831 | 0.0069 | **0.0111** |
|  | Participant’s Organization Category: Provincial | - | - | - | **-** | **-** | **-** |
|  | Participant’s Organization Category: Indigenous | -1.0612 | 0.3187 | -0.1124 | -0.1087 | 0.0118 | **0.0009** |
|  | Participant’s Organization Category: Research | 0.1757 | 0.1059 | 0.0828 | 0.0542 | 0.0029 | 0.0974 |
|  | Participant’s Organization Category: Fishers | -0.2194 | 0.1295 | -0.0707 | -0.0553 | 0.0031 | 0.0906 |
| 2 | Internal Efficacy (IE) | -0.0291 | 0.0332 | -0.0357 | -0.0286 | 0.0008 | 0.3812 |
| 3 | Criterion-scaled Participants | 1.0000 | 0.0343 | 0.7222 | 0.6791 | 0.4611 | **0.0000** |
| 4 | Target Organization Category: Regional | 0.2316 | 0.1113 | 0.0775 | 0.0468 | 0.0022 | **0.0378** |
|  | Target Organization Category: CA Federal | -0.2108 | 0.1011 | -0.0684 | -0.0469 | 0.0022 | **0.0375** |
|  | Target Organization Category: US Federal | -0.0567 | 0.0987 | -0.0285 | -0.0129 | 0.0002 | 0.5659 |
|  | Target Organization Category: State | 0.0074 | 0.1022 | 0.0035 | 0.0016 | 0.0000 | 0.9425 |
|  | Target Organization Category: Provincial | - | - | - | - | - | - |
|  | Target Organization Category: Indigenous | -0.1507 | 0.1302 | -0.0316 | -0.0261 | 0.0007 | 0.2473 |
|  | Target Organization Category: NGO | -0.3096 | 0.1049 | -0.1309 | -0.0664 | 0.0044 | **0.0033** |
|  | Target Organization Category: Research | -0.1754 | 0.1028 | -0.0809 | -0.0384 | 0.0015 | 0.0884 |
|  | Target Organization Category: Fishers | -0.2857 | 0.1020 | -0.1362 | -0.0631 | 0.0040 | **0.0052** |
| 5 | Legitimacy Dependence (LEGD) | 0.1713 | 0.0209 | 0.2072 | 0.1775 | 0.0315 | **0.0000** |
| 6 | Capital Dependence (CAPD) | 0.1290 | 0.0217 | 0.1372 | 0.1266 | 0.0160 | **0.0000** |
| 7 | Regulatory Dependence (REGD) | 0.0662 | 0.0172 | 0.1058 | 0.0812 | 0.0066 | **0.0001** |
| 8 | Sanction Risk (SR) | 0.0459 | 0.0232 | 0.0531 | 0.0418 | 0.0017 | **0.0480** |
| 9 | Performance Risk (PR) | -0.2005 | 0.0237 | -0.2057 | -0.1718 | 0.0295 | **0.0000** |
| 10 | SR × IE | 0.0509 | 0.0181 | 0.0607 | 0.0566 | 0.0032 | **0.0051** |
|  | SR × LEGD | 0.0071 | 0.0211 | 0.0084 | 0.0068 | 0.0000 | 0.7350 |
|  | SR × CAPD | 0.0439 | 0.0221 | 0.0479 | 0.0399 | 0.0016 | **0.0478** |
|  | SR × REGD | 0.0154 | 0.0168 | 0.0237 | 0.0185 | 0.0003 | 0.3584 |
| 11 | PR × IE | -0.0254 | 0.0235 | -0.0248 | -0.0216 | 0.0005 | 0.2808 |
|  | PR × LEGD | 0.0265 | 0.0207 | 0.0310 | 0.0256 | 0.0007 | 0.2015 |
|  | PR × CAPD | 0.0686 | 0.0266 | 0.0629 | 0.0518 | 0.0027 | **0.0100** |
|  | PR × REGD | -0.0409 | 0.0183 | -0.0582 | -0.0447 | 0.0020 | 0.0262 |

^a^ Dependent variable: External efficacy. Note. Bold font gives us statistically significant predictors and values.

**Table S2**: Hierarchical Regression Analysis: Goal **Conflict**

| Hierarchical regression model summary ^a^ | | | | | | | |
| --- | --- | --- | --- | --- | --- | --- | --- |
|  |  | R^2^ change statistics | | | | | |
| Model | Predictor set entered | Model R^2^ | R^2^ Change | *df p*redictors | *df* residual | F-test | P |
| 1 | Participant’s Organization Category | 0.0967 | 0.0967 | 7 | 882 | 13.49 | **0.0000** |
| 2 | Internal Efficacy (IE) | 0.0975 | 0.0008 | 8 | 881 | 11.90 | **0.0000** |
| 3 | Criterion-scaled Participants | 0.5377 | 0.4402 | 9 | 880 | 113.72 | **0.0000** |
| 4 | Target Organization Category | 0.5819 | 0.0442 | 17 | 872 | 71.38 | **0.0000** |
| 5 | Legitimacy Dependence (LEGD) | 0.5917 | 0.0098 | 18 | 871 | 70.11 | **0.0000** |
| 6 | Capital Dependence (CAPD) | 0.5941 | 0.0024 | 19 | 870 | 67.02 | **0.0000** |
| 7 | Regulatory Dependence (REGD) | 0.5943 | 0.0002 | 20 | 869 | 63.64 | **0.0000** |
| 8 | Sanction Risk (SR) | 0.5970 | 0.0027 | 21 | 868 | 61.23 | **0.0000** |
| 9 | Performance Risk (PR) | 0.6446 | 0.0476 | 22 | 867 | 71.49 | **0.0000** |
| 10 | SR Interactions (SR×IE, SR×LEGD, SR×CAPD, and SR×REGD) | 0.6487 | 0.0041 | 26 | 863 | 61.30 | **0.0000** |
| 11 | PR Interactions (PR×IE, PR×LEGD, PR×CAPD, and PR×REGD) | 0.6552 | 0.0065 | 30 | 859 | 54.41 | **0.0000** |
| 12 | External Efficacy (EE) | 0.6575 | 0.0023 | 31 | 858 | 53.14 | **0.0000** |
| Model coefficients | |  |  |  |  |  |  |
|  |  | Unstandardized  coefficients | | Standardized  coefficients | Correlations | | F-test |
| Model | Individual predictor | β | Std. error | β | Part | sr^2^ | P |
| 1 | Participant’s Organization Category: Regional | -0.3476 | 0.1100 | -0.1575 | -0.1012 | 0.0102 | **0.0016** |
|  | Participant’s Organization Category: CA Federal | -0.6068 | 0.1339 | -0.1874 | -0.1450 | 0.0210 | **0.0000** |
|  | Participant’s Organization Category: US Federal | -0.7484 | 0.1019 | -0.4089 | -0.2349 | 0.0552 | **0.0000** |
|  | Participant’s Organization Category: State | -0.8833 | 0.1126 | -0.3793 | -0.2510 | 0.0630 | **0.0000** |
|  | Participant’s Organization Category: Provincial | - | - | - | **-** | **-** | **-** |
|  | Participant’s Organization Category: Indigenous | 0.3059 | 0.3357 | 0.0301 | 0.0292 | 0.0009 | 0.3624 |
|  | Participant’s Organization Category: Research | -0.5393 | 0.1116 | -0.2365 | -0.1547 | 0.0239 | **0.0000** |
|  | Participant’s Organization Category: Fishers | -0.5387 | 0.1364 | -0.1614 | -0.1264 | 0.0160 | **0.0001** |
| 2 | Internal Efficacy (IE) | 0.0317 | 0.0350 | 0.0362 | 0.0290 | 0.0008 | 0.3650 |
| 3 | Criterion-scaled Participants | 1.0000 | 0.0345 | 0.7333 | 0.6635 | 0.4402 | **0.0000** |
| 4 | Target Organization Category: Regional | -0.2366 | 0.1165 | -0.0736 | -0.0445 | 0.0020 | **0.0425** |
|  | Target Organization Category: CA Federal | -0.0065 | 0.1059 | -0.0019 | -0.0013 | 0.0000 | 0.9514 |
|  | Target Organization Category: US Federal | -0.0942 | 0.1033 | -0.0441 | -0.0200 | 0.0004 | 0.3621 |
|  | Target Organization Category: State | -0.1503 | 0.1069 | -0.0659 | -0.0308 | 0.0009 | 0.1600 |
|  | Target Organization Category: Provincial | - | - | - | - | - | - |
|  | Target Organization Category: Indigenous | 0.1129 | 0.1363 | 0.0220 | 0.0181 | 0.0003 | 0.4075 |
|  | Target Organization Category: NGO | 0.3371 | 0.1098 | 0.1326 | 0.0672 | 0.0045 | **0.0022** |
|  | Target Organization Category: Research | -0.0659 | 0.1075 | -0.0283 | -0.0134 | 0.0002 | 0.5399 |
|  | Target Organization Category: Fishers | 0.2228 | 0.1066 | 0.0988 | 0.0458 | 0.0021 | **0.0369** |
| 5 | Legitimacy Dependence (LEGD) | -0.0991 | 0.0217 | -0.1115 | -0.0989 | 0.0098 | **0.0000** |
| 6 | Capital Dependence (CAPD) | -0.0541 | 0.0237 | -0.0535 | -0.0494 | 0.0024 | **0.0225** |
| 7 | Regulatory Dependence (REGD) | -0.0118 | 0.0190 | -0.0175 | -0.0134 | 0.0002 | 0.5358 |
| 8 | Sanction Risk (SR) | 0.0637 | 0.0264 | 0.0685 | 0.0520 | 0.0027 | **0.0159** |
| 9 | Performance Risk (PR) | 0.2736 | 0.0254 | 0.2611 | 0.2183 | 0.0476 | **0.0000** |
| 10 | SR × IE | -0.0422 | 0.0195 | -0.0469 | -0.0437 | 0.0019 | **0.0305** |
|  | SR × LEGD | -0.0373 | 0.0227 | -0.0411 | -0.0330 | 0.0011 | 0.1018 |
|  | SR × CAPD | 0.0145 | 0.0239 | 0.0147 | 0.0122 | 0.0001 | 0.5440 |
|  | SR × REGD | -0.0163 | 0.0182 | -0.0233 | -0.0181 | 0.0003 | 0.3698 |
| 11 | PR × IE | 0.0299 | 0.0253 | 0.0272 | 0.0237 | 0.0006 | 0.2371 |
|  | PR × LEGD | -0.0477 | 0.0222 | -0.0521 | -0.0430 | 0.0018 | **0.0321** |
|  | PR × CAPD | -0.0659 | 0.0285 | -0.0562 | -0.0463 | 0.0021 | **0.0211** |
|  | PR × REGD | 0.0059 | 0.0197 | 0.0078 | 0.0060 | 0.0000 | 0.7657 |
| 12 | External Efficacy (EE) | -0.0646 | 0.0268 | -0.0601 | -0.0481 | 0.0023 | **0.0162** |

^a^ Dependent variable: Conflict. Note. Bold font gives us statistically significant predictors and values.
